# Echocardiography as a Vital Tool in Assessing Shock: A Comprehensive Review

**DOI:** 10.7759/cureus.57310

**Published:** 2024-03-31

**Authors:** Abhishek Jain, Amol Singam, V. N. K. Srinivas Mudiganti

**Affiliations:** 1 Critical Care Medicine, Jawaharlal Nehru Medical College, Datta Meghe Institute of Higher Education and Research, Wardha, IND

**Keywords:** management, diagnosis, hemodynamics, assessment, echocardiography, shock

## Abstract

Shock is a critical condition characterized by inadequate tissue perfusion, leading to cellular hypoxia and organ dysfunction. Early and accurate assessment is crucial for timely intervention and improved patient outcomes. Echocardiography has emerged as a vital tool in the assessment of shock, offering real-time visualization of cardiac anatomy, function, and hemodynamics. This comprehensive review aims to elucidate the role of echocardiography in shock assessment by providing an overview of its principles, techniques, and clinical applications. We discuss the importance of early diagnosis, identification of underlying pathology, monitoring response to therapy, and prognostic value offered by echocardiography in managing shock. Furthermore, we explore its utility in different types of shock, including hypovolemic, cardiogenic, distributive, and obstructive shock. Challenges and limitations of echocardiography, as well as future directions and innovations, are also discussed. Through a synthesis of current evidence and clinical insights, this review underscores the significance of echocardiography in optimizing shock management and highlights areas for further research and development.

## Introduction and background

Shock is a critical medical condition characterized by inadequate tissue perfusion, resulting in cellular hypoxia and organ dysfunction. It is a life-threatening state that requires prompt intervention to prevent irreversible damage or death [1]. Early recognition and prompt intervention are paramount in managing shock. Timely assessment allows healthcare providers to identify the underlying cause, initiate appropriate treatment, and prevent further deterioration. Accurate assessment aids in optimizing patient outcomes and reducing mortality rates associated with shock [2].

Echocardiography plays a crucial role in the assessment of shock by providing real-time visualization of cardiac anatomy, function, and hemodynamics. It offers valuable insights into the underlying etiology of shock, aids in identifying specific cardiac abnormalities contributing to hemodynamic instability and guides targeted therapy [3]. This review aims to provide a comprehensive overview of the role of echocardiography in assessing shock. The aim of the review is to enhance understanding and promote the optimal utilization of echocardiography in managing patients with shock by synthesizing current evidence and discussing clinical applications, limitations, and future directions.

## Review

Echocardiography: principles and techniques

Basics of Echocardiography

Echocardiography, commonly referred to as echo, represents a non-invasive medical imaging technique that employs ultrasound waves to assess the heart's structure and function. Its evolution over time has positioned it as a primary choice for cardiac evaluation owing to its efficacy and safety [4-6]. At its core, echocardiography utilizes ultrasound waves to generate images of the heart's internal structures through the emission and detection of reflected sound waves at tissue interfaces [5,6]. Various echocardiographic modes, including two-dimensional (2D) imaging, M-mode imaging, and Doppler imaging, are employed to visualize the heart [5].

The predominant mode for echocardiography is two-dimensional (2D) imaging, which permits real-time visualization of cardiac structures in cross-sections such as the parasternal long axis, parasternal short axis, and apical views [5]. M-mode imaging offers precise measurements of cardiac structures with high temporal and spatial resolutions, focusing on a single line from the 2D trace [5]. Grounded in the Doppler effect, Doppler imaging enables blood flow velocity assessment by analyzing frequency changes between transmitted and reflected sound waves, thus providing valuable insights into cardiac hemodynamics [5].

Echocardiography is critical in diagnosing various cardiac conditions by evaluating heart size, valve function, wall thickness, blood flow patterns, and more [5,6]. It proves instrumental in assessing systolic and diastolic function, detecting abnormalities such as hypertrophy, heart failure, and pericarditis, and estimating pressures within the heart chambers [4,6]. With new technologies like Doppler and three-dimensional (3D) imaging, echocardiography has progressed significantly, augmenting its diagnostic capabilities and solidifying its status as an indispensable tool in contemporary cardiology [5,6].

Different Modalities

Transthoracic echocardiography (TTE): TTE is a cornerstone imaging modality in cardiac assessment, offering a non-invasive or minimally invasive means to visualize the heart's structures and functions utilizing ultrasound technology [7,8]. This technique entails positioning a transducer on the chest wall to capture images from standardized windows such as the left parasternal, apical, subcostal, and suprasternal notch, facilitating a comprehensive evaluation of the heart's chambers, valves, and blood vessels [7,9]. TTE is critical in diagnosing various cardiac conditions, assessing heart health, pinpointing causes of symptoms, and screening for specific medical conditions like hypertrophy, heart failure, and valvular abnormalities [7,9]. Furthermore, TTE plays a pivotal role in evaluating systolic and diastolic function, identifying abnormalities such as hypertrophic cardiomyopathy, restrictive cardiomyopathy, severe heart failure, and constrictive pericarditis [8]. This modality furnishes valuable insights into heart wall thickness, motion, ischemia, infarction, valvular function, intracardiac thrombus, pulmonary arterial pressure, and central venous pressure [8]. Moreover, TTE can estimate intracardiac pressures and assess left ventricular hypertrophy through diastolic filling patterns [8].

Transesophageal echocardiography (TEE): TEE represents a specialized imaging examination employing ultrasound technology to generate detailed heart images by inserting a probe down the esophagus. This technique offers clearer and more precise visualization of the heart's chambers, valves, blood vessels, and outer lining compared to conventional echocardiograms conducted on the chest's surface [10]. TEE proves particularly advantageous in evaluating specific heart conditions such as mitral valve disorders, intracardiac blood clots or masses, aortic dissections, and the structure and function of prosthetic heart valves [10]. During a TEE procedure, a transducer emits ultrasonic waves that reflect off heart structures, subsequently captured by a computer to produce images of the heart's walls and valves. This approach yields invaluable insights into cardiac function and pathology, facilitating the diagnosis of various heart conditions, including congestive heart failure, cardiomyopathy, valvular heart disease, aneurysms, pericarditis, endocarditis, and intracardiac thrombi [10]. TEE is minimally invasive and generally well-tolerated, with potential risks including bleeding, respiratory complications, or arrhythmias [11]. Patients undergoing TEE procedures typically receive sedation to ensure comfort during the examination. Pre-procedural preparations include fasting for several hours, disclosing medications and allergies to healthcare providers, and arranging transportation due to sedation-induced effects [11]. Following the procedure, patients may experience mild discomfort, such as a sore throat, but can usually resume normal activities within 24 hours post-procedure [11].

Interpretation of Echocardiographic Findings

Interpreting echocardiographic findings involves systematically extracting clinically relevant information from the report to guide clinical decision-making effectively. Echocardiography yields many structural and functional data, making it imperative to discern findings directly impacting patient care [12]. Physicians' primary objective is to identify echocardiographic findings with significant implications for clinical outcomes, such as left ventricular function, valvular abnormalities, and hemodynamic status [12]. When interpreting an echocardiography report, attention should be paid to the study's clinical indication, the echocardiography type (transthoracic or transesophageal), hemodynamics during the study, and image quality [12]. Image quality is crucial for accurate diagnosis, and suboptimal images may necessitate additional modalities like ultrasound contrast or transesophageal echocardiography to overcome limitations. To streamline interpretation, tables, and structured approaches are often employed to summarize key echocardiographic findings influencing clinical decision-making, facilitating the extraction of pertinent information from the report. By comprehending the significance of diverse echocardiographic parameters, physicians can make informed decisions regarding patient management, further diagnostic assessments, and treatment strategies based on the report's findings [13].

Role of echocardiography in assessing shock

Early Diagnosis and Hemodynamic Assessment

In critical care settings, patients' timely diagnosis and hemodynamic assessment are paramount in guiding appropriate treatment strategies and ultimately improving patient outcomes. Recent research has shed light on the critical role of early hemodynamic assessment, particularly in elderly patients admitted to medical ICUs, underscoring the significance of prompt evaluation and its consequential impact on treatment decisions [14]. These studies emphasize that hemodynamic assessments, including transthoracic echocardiography and continuous invasive monitoring, are indispensable tools for managing elderly patients with acute conditions, leading to therapeutic modifications and enhanced outcomes [14]. Moreover, investigations into the utilization of non-invasive cardiac output monitoring (NICOM) for early hemodynamic assessment in patients at risk of sepsis have yielded valuable insights into the predictive capabilities of serial hemodynamic parameters obtained from this device [15]. While studies have demonstrated that cardiac output (CO) and cardiac index (CI) measured at different time points may not facilitate early differentiation of disease severity in patients at risk for severe sepsis, serum lactate levels have emerged as a superior predictor of patient admission from the emergency department [15]. These findings underscore the importance of integrating various hemodynamic parameters and laboratory markers like lactate to augment early diagnosis and risk stratification in critically ill patients.

Identification of Underlying Pathology

Identifying underlying pathology in patients experiencing shock is pivotal for directing appropriate management strategies. Echocardiography emerges as a valuable tool in this endeavor by enabling the early detection of chronic underlying pathologies, facilitating a shift towards quantitative assessment and tailored treatment plans [16]. This imaging modality furnishes detailed insights into cardiac function, structural abnormalities, and hemodynamic parameters, thereby aiding in the prompt diagnosis and characterization of various conditions contributing to shock states [16,17]. Through echocardiography, clinicians can pinpoint specific cardiac pathologies such as valvular diseases, myocardial dysfunction, and fluid overload, thus facilitating targeted interventions to address the root cause of shock and enhance patient outcomes [17,18]. Moreover, echocardiography is significant in evaluating mitral valve disease by comprehensively assessing mitral stenosis and regurgitation through qualitative, quantitative, and semi-quantitative evaluations [18]. This meticulous assessment encompasses examining mitral valve leaflets, subvalvular apparatus, left ventricular size, ejection fraction, wall motion abnormalities, and other parameters crucial for determining the severity of valvular lesions and planning appropriate therapeutic strategies [18]. The integration of various echocardiographic modalities such as 2D echo, 3D echo, and stress echo further enhances diagnostic accuracy and aids in defining the grade of severity in conditions like mitral regurgitation [18].

Monitoring Response to Therapy

The role of echocardiography in monitoring the response to therapy holds critical significance across various medical conditions, notably heart failure and pulmonary hypertension. Echocardiography is a valuable tool for evaluating the effectiveness of treatments and informing further management decisions based on observed changes in cardiac function and structure. In patients with heart failure, echocardiography is instrumental in monitoring the response to therapies such as high doses of ACE inhibitors, spironolactone, and beta-blockers. This monitoring entails assessing clinical symptoms, diastolic function, and survival rates over treatment to gauge the therapy's impact on symptom improvement and cardiac function [19]. Regular echocardiographic assessments enable healthcare providers to monitor changes in ejection fraction, ventricular dimensions, and diastolic function, thereby offering valuable insights into the patient's response to treatment and guiding adjustments in therapy as necessary. Similarly, in pulmonary hypertension, echocardiography plays a vital role in monitoring treatment response by evaluating the morphological and functional consequences of the condition. Echocardiographic parameters such as tricuspid annular plane excursion, right atrial enlargement, and pericardial effusion indicate disease severity and prognosis. It is recommended to perform echocardiographic monitoring every 3-4 months following the initiation or modification of therapy to assess treatment efficacy and guide further management strategies based on observed changes in cardiac parameters [20].

Prognostic Value

**Figure 1:**
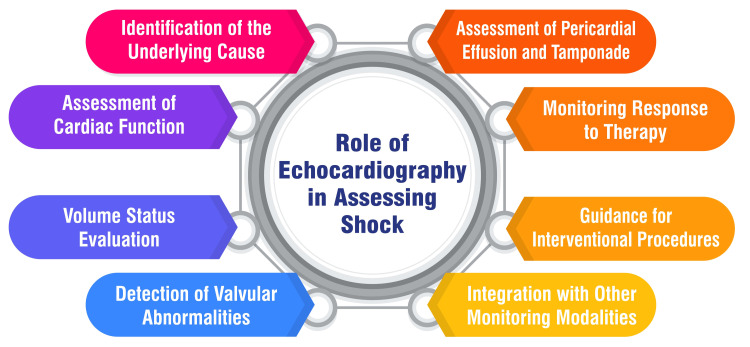
Role of echocardiography in assessing shock The image is created by the corresponding author.

The prognostic value of echocardiography in various medical conditions is substantial, as evidenced by numerous studies. Echocardiographic parameters, particularly those about right ventricular dysfunction (RVD), have emerged as valuable predictors of outcomes across different patient populations. In normotensive patients with acute pulmonary embolism (APE), echocardiographic indices of RVD, such as tricuspid annulus plane systolic excursion (TAPSE), have been identified as independent predictors of adverse events, including APE-related mortality and the need for thrombolysis [21]. TAPSE values offer superior predictive value compared to the right ventricular (RV)/left ventricular (LV) ratio, with TAPSE ≤15 mm indicating heightened risk and TAPSE >20 mm identifying a low-risk group [21]. Similarly, echocardiography has been assessed for its prognostic value in hypertensive individuals. Studies indicate that echocardiographic findings can provide additional prognostic information beyond traditional risk factors, facilitating risk stratification and guiding management decisions for hypertensive patients [22]. Furthermore, echocardiography is pivotal in both diagnosis and prognosis in the context of pulmonary hypertension (PH). Various echocardiographic parameters, including right ventricular ejection fraction, tricuspid annular plane systolic excursion (TAPSE), and RV strain measured by speckle tracking echocardiography, have been identified as valuable prognostic markers for PH patients, reflecting disease severity and predicting outcomes [23]. Figure 1 shows the role of echocardiography in assessing shock.

Echocardiography in different types of shock

Hypovolemic Shock

Hypovolemic shock represents a life-threatening condition marked by a critical depletion in the effective circulating blood volume, precipitating systemic hypoperfusion and tissue hypoxia. If left unaddressed, hypovolemic shock can lead to ischemic injury in vital organs, multi-system organ failure, and, ultimately, death [24,25]. Managing hypovolemic shock involves swiftly identifying the cause of volume loss, whether from total body fluid depletion or hemorrhage and initiating blood or fluid replacement to mitigate tissue ischemia [25]. Factors such as the nature and pace of fluid replacement are pivotal considerations in hypovolemic shock treatment, aiming to restore euvolemia and avert further complications [25]. Echocardiography is significant in diagnosing and managing hypovolemic shock by furnishing a non-invasive and rapid assessment of cardiac function and hemodynamics in shocked patients [3]. This imaging modality empowers clinicians to pinpoint specific echocardiographic features associated with hypovolemic shock, such as reduced cardiac cavity size, diminished left heart filling pressures, decreased aortic velocity time integral, and cardiac output [3,26]. Leveraging echocardiography, healthcare providers can differentiate hypovolemic shock from other shock types, tailor appropriate treatment strategies, and effectively monitor the response to therapies [3,26].

Cardiogenic Shock

Cardiogenic shock represents a severe condition characterized by a notable impairment of myocardial performance, resulting in hemodynamic instability and insufficient tissue perfusion. It continues to be associated with elevated morbidity and mortality rates, particularly among patients admitted to cardiac intensive care units [27]. Echocardiography is crucial in managing cardiogenic shock by facilitating the prompt recognition of this condition, identifying underlying etiologies, and assessing the severity of hemodynamic dysfunction to devise accurate treatment strategies [28]. A study comparing transthoracic echocardiography (TTE) findings in patients experiencing cardiogenic shock due to acute myocardial infarction (AMI-CS) versus heart failure (HF-CS) unveiled distinct differences in clinical and echocardiographic parameters between the two cohorts [29]. Individuals with AMI-CS exhibited superior left ventricular function, lower biventricular filling pressures, and higher stroke volume than those with HF-CS. Notably, myocardial contraction fraction emerged as a valuable prognostic parameter for predicting mortality in AMI-CS patients [29]. The utilization of echocardiography in ascertaining the prognosis of patients admitted with AMI-CS underscores its significance in early triage and guiding targeted therapy for individuals experiencing cardiogenic shock [29]. By leveraging echocardiographic assessments, clinicians can tailor treatment approaches based on specific cardiac parameters, thereby contributing to improved outcomes and enhanced management strategies for patients grappling with cardiogenic shock.

Distributive Shock

Distributive shock, or vasodilatory shock, is a critical medical condition characterized by systemic vasodilation, leading to diminished blood flow to vital organs such as the brain, heart, and kidneys. This state results in inadequate tissue perfusion and can damage organs due to reduced blood flow. Unlike other types of shock, distributive shock involves abnormal blood flow distribution in the smallest blood vessels, even when sufficient oxygen-carrying blood is available [30,31]. Common causes of distributive shock encompass sepsis, anaphylaxis, neurogenic shock, and adrenal crisis. Sepsis, the most prevalent cause, often arises from an immune response dysregulation triggered by infection, precipitating systemic inflammation and vasodilation. Anaphylactic shock stems from severe allergic reactions, whereas neurogenic shock results from the loss of sympathetic nervous system support due to injury or trauma.

Additionally, adrenal crisis can induce distributive shock due to inadequate steroid hormone production [30,31]. The diagnosis of distributive shock entails recognizing symptoms such as rapid breathing (tachypnea), fast heart rate (tachycardia), low blood pressure (hypotension), altered mental status, and fever. Treatment aims to reverse the underlying cause and achieve hemodynamic stabilization through fluid resuscitation and administering vasoactive drugs like vasopressors and inotropes. Specific interventions may involve administering antibiotics for septic shock and beta-sympathomimetics for select cases [30,31].

Obstructive Shock

Obstructive shock presents as a life-threatening condition characterized by inadequate tissue oxygen supply stemming from diminished cardiac output due to noncardiac diseases. This shock arises from physical impediments obstructing blood flow, reducing venous return, heightened afterload, and decreased cardiac output [32]. Common causes of obstructive shock encompass pulmonary embolism, tension pneumothorax, pericardial tamponade, and aortic dissection [33]. Diagnosis of obstructive shock entails a structured approach, including clinical ultrasound examination utilizing the rapid ultrasound in shock (RUSH) protocol and radiological imaging if deemed necessary [33]. It is critical to distinguish obstructive shock from cardiogenic shock, as the treatment strategies vary significantly. While cardiogenic shock results from primary cardiac dysfunction, obstructive shock arises from diminished venous return or increased afterload unrelated to cardiac function [32]. Treatment for obstructive shock focuses on promptly addressing the underlying cause. For instance, tension pneumothorax necessitates swift needle decompression to restore blood flow to and from the heart, alleviating obstruction and reversing shock [33]. Fluid therapy alone proves inadequate in reversing obstructive shock, underscoring the importance of identifying and treating the specific cause. In scenarios such as cardiac tamponade or severe pulmonary embolism, immediate interventions like pericardiocentesis or thrombolysis may be lifesaving [32].

Challenges and limitations of echocardiography in shock assessment

Technical Challenges

The technical challenges associated with echocardiography in the assessment of shock are underscored in the study, elucidating the diverse roles of echocardiography in shock treatment [34]. One of the paramount challenges lies in the necessity for skilled operators to ensure precise image acquisition and interpretation, given the critical role of echocardiographic image quality in diagnostic accuracy, particularly in emergency settings where swift and accurate diagnosis is imperative [35]. Moreover, technical obstacles arise from time constraints and patient-related factors such as obesity or suboptimal echo views, which can impede the efficacy of echocardiography in swiftly identifying the underlying cause of shock [36]. Additionally, the differentiation between certain types of shock using echocardiography poses a challenge, particularly in scenarios where distributive shocks like hypovolemic shock and early septic shock may exhibit analogous findings in imaging studies [37]. This limitation underscores the complexity of precisely identifying the etiology of shock solely based on echocardiographic findings, necessitating a comprehensive approach incorporating other clinical parameters and diagnostic modalities [38]. While echocardiography is a valuable tool in assessing shock, addressing these technical challenges is pivotal to optimizing its diagnostic utility and ensuring timely and accurate management of patients in shock states.

Patient Factors

Patient factors play a pivotal role in the assessment and management of shock. Various aspects of a patient's condition, encompassing vital signs, clinical presentation, medical history, and response to treatment, significantly influence the approach to shock. In the context of shock assessment, indicators such as altered mental status, tachycardia, hypotension, oliguria, and skin appearance serve as critical markers guiding clinicians in diagnosing and categorizing shock [39]. Moreover, factors like temperature, heart rate, blood pressure, oxygen saturation, and response to fluid resuscitation constitute essential considerations in evaluating a patient's hemodynamic status and determining the appropriate interventions [40,41]. Beyond the initial assessment, patient factors extend to ongoing management strategies for shock. Considerations such as trauma, fluid responsiveness, risk of fluid overload, temperature regulation, vasopressor requirements, and the potential need for blood product administration are critical in tailoring treatment to address the underlying cause of shock and optimize patient outcomes [42]. Understanding these patient-specific factors empowers healthcare providers to individualize care, monitor response to interventions, and adapt treatment strategies to enhance hemodynamic stability and overall prognosis in patients grappling with shock.

Interpretation Challenges

**Figure 2:**
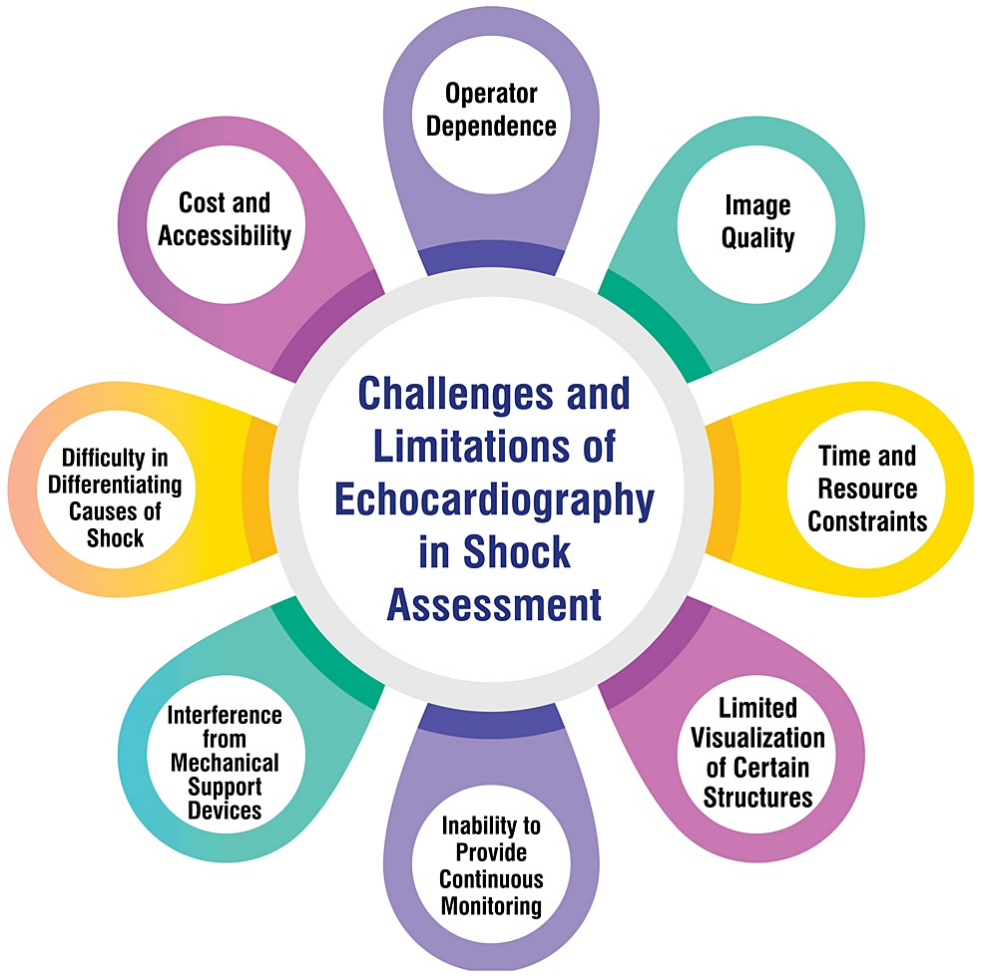
Challenges and limitations of echocardiography in shock assessment The image is created by the corresponding author.

Novice trainees encounter challenges in interpreting transthoracic echocardiograms (TTEs) due to their low diagnostic accuracy and lack of a systematic approach in their training [43]. In contrast, experts and advanced fellows utilize specific techniques such as previewing studies, reviewing multiple images simultaneously, and maintaining flexibility in the order of image review, which novices may need to employ, resulting in differences in interpretation efficiency [43]. Students embarking on echocardiography education confront difficulty in probe handling, understanding projections, and correlating the transducer with the heart's anatomy [44]. Overcoming these initial learning hurdles is facilitated by immediate feedback, practical experience with the ultrasound machine, video lectures, and the opportunity to learn from mistakes in a risk-free environment [44]. Non-cardiologists and individuals outside the imaging field often need help interpreting echocardiography reports due to the dynamic nature of the field, evolving imaging modalities, and varying reporting formats across laboratories [45]. This complexity challenges effective clinical decision-making based on echocardiographic findings, highlighting the necessity for a systematic approach to extract clinically relevant information from reports [45]. Integrating deep learning in interpreting echocardiograms introduces challenges associated with accurately predicting anatomic structures, local features, and systemic risk factors from images [46]. While machine learning models hold the potential to streamline repetitive tasks and offer preliminary interpretations, ensuring the robustness and precision of these interpretations remains a critical challenge in clinical practice [46]. Figure 2 shows the challenges and limitations of echocardiography in shock assessment.

Future directions and innovations

Advancements in Echocardiographic Technology

Advanced echocardiographic technology has profoundly revolutionized cardiac imaging, offering various innovative features that augment diagnostic capabilities and patient care. Echocardiography has progressed from basic M-mode tracing to encompass a sophisticated array of technologies, including two-dimensional imaging, spectral Doppler, color flow Doppler, tissue Doppler, and transesophageal echocardiography [47]. The shift from analog to digital signal processing has transformed ultrasound imaging, resulting in enhanced image resolution, compact instrumentation for bedside evaluation, and digital image storage for precise quantification and comparison with prior studies [47]. Recent strides in echocardiography technology entail the advent of three-dimensional (3D) imaging capabilities, presenting refined techniques for chamber quantification and innovative visualization methods for cardiac valves such as 3D printing, virtual reality, and holography [48]. These advancements broaden the scope of echocardiography by enabling a comprehensive evaluation of cardiac structures in their entirety, thereby enhancing diagnostic accuracy and facilitating treatment planning [48]. Furthermore, integrating artificial intelligence (AI) into echocardiography represents another significant breakthrough already being integrated into clinical practice to enhance image acquisition, interpretation, workflow efficiency, and patient throughput [49]. AI applications in cardiac ultrasound seek to automate tasks, optimize diagnostic processes, and bolster the reproducibility of measurements, ultimately advancing patient care within the realm of cardiology [49].

Integration with Other Monitoring Tools

Integrating echocardiography with other monitoring tools is a pivotal aspect of augmenting its utility and efficiency in clinical practice. This integration empowers healthcare providers to conduct a more comprehensive cardiac function and hemodynamics assessment, ultimately improving patient care outcomes. One critical facet of integration involves incorporating artificial intelligence (AI) into echocardiography systems. AI-based software can facilitate image measurements, automate the quantification of echocardiograms, and transform the interpretation of echocardiographic images, thereby enabling more efficient and accurate diagnosis and monitoring of cardiac conditions [50]. This integration with AI enhances echocardiography's diagnostic capabilities, streamlines workflow, and enhances the overall efficiency of cardiac imaging services [50]. Moreover, the seamless integration of echocardiography with advanced post-processing tools, such as the EchoPAC Suite, facilitates in-depth analysis, measurements, and reporting of echocardiographic datasets [51]. These tools empower clinicians to review, analyze, and perform measurements offline, enhancing workflow efficiency and ensuring patient information is stored in a centralized location for easy access and reference [51].

Potential Areas for Research and Development

Continued evolution in real-time three-dimensional (RT3D) imaging techniques drives advancements in 3-dimensional echocardiography, offering enhanced visualization of cardiac structures and functions. These developments promise to facilitate more accurate diagnoses and treatment planning [52]. Integrating artificial intelligence (AI) into echocardiography presents significant opportunities to revolutionize clinical workflows, optimize image acquisition, and augment diagnostic accuracy. By leveraging AI, healthcare providers can streamline processes, leading to more efficient patient care and increased throughput [49]. The field of interventional echocardiography is experiencing rapid expansion, particularly in guiding various transcatheter procedures such as valve repairs, replacements, and other structural heart interventions. This growth offers patients minimally invasive treatment options and advanced imaging guidance, marking a significant stride in cardiac intervention [49]. The utilization of point-of-care echocardiography across diverse settings, including pediatrics, interventional procedures, and new disease identification, is reshaping the role of echocardiography. By making echocardiography faster, more reproducible, and less operator-dependent, point-of-care applications enhance patient care delivery [49]. Addressing the challenge of funding and reimbursement for novel echocardiography technologies is paramount to ensure patient access to innovative advancements. Advocacy efforts are crucial in supporting the adoption of cutting-edge echocardiographic tools and promoting equitable access to advanced cardiac imaging technologies [49].

## Conclusions

In conclusion, echocardiography emerges as an indispensable tool in assessing and managing shock. Throughout this review, its pivotal role has been underscored, serving as a non-invasive, bedside modality that provides critical insights into cardiac function, hemodynamics, and the underlying etiology of shock. By enabling early diagnosis, guiding targeted therapy, and facilitating real-time monitoring of patient response, echocardiography significantly enhances the accuracy of shock assessment, leading to improved outcomes and reduced mortality rates. Its integration into routine clinical practice is paramount, necessitating proficiency among healthcare providers in both performing and interpreting echocardiograms. Moreover, collaboration among multidisciplinary teams is vital for seamlessly integrating echocardiography into shock management protocols. Looking ahead, further research is warranted to validate the prognostic value of echocardiographic parameters, explore the impact on healthcare resource utilization, and leverage advancements in echocardiographic techniques for refined risk stratification and therapeutic guidance in managing shock. Overall, echocardiography is a cornerstone in the armamentarium of critical care, offering invaluable insights and driving improvements in patient care and outcomes.
